# In vitro analysis of shear bond strength and adhesive remnant index of different metal brackets

**DOI:** 10.1590/2177-6709.21.6.067-073.oar

**Published:** 2016

**Authors:** Fernanda de Souza Henkin, Érika de Oliveira Dias de Macêdo, Karoline da Silva Santos, Marília Schwarzbach, Susana Maria Werner Samuel, Karina Santos Mundstock

**Affiliations:** 1 Orthodontics’ graduate student, Pontifícia Universidade Católica do Rio Grande do Sul, Porto Alegre/RS, Brazil.; 2 Professor of Certificate in Orthodontic Program, Orthodontics Department, Universidade Federal do Rio Grande do Sul, Porto Alegre/RS, Brazil.; 3 Undergraduate student, School of Dentistry, Universidade Federal do Rio Grande do Sul, Porto Alegre/RS, Brazil.; 4 Orthodontic Certificate student, Orthodontics Department, Universidade Federal do Rio Grande do Sul, Porto Alegre/RS, Brazil.; 5 Titular Professor, Dental Materials Laboratory, School of Dentistry, Universidade Federal do Rio Grande do Sul, Porto Alegre/RS.; 6 Adjunct Professor, Orthodontics Department, School of Dentistry, Universidade Federal do Rio Grande do Sul, Porto Alegre/RS, Brazil.

**Keywords:** Shear strength, Orthodontic brackets, Orthodontics

## Abstract

**Introduction:**

There is a great variety of orthodontic brackets in the Brazilian market, and constantly evaluating them is critical for professionals to know their properties, so as to be able to choose which product best suits their clinical practice.

**Objectives:**

To evaluate the bond strength and the adhesive remnant index (ARI) of different brands of metal brackets.

**Material and Methods:**

A total of 105 bovine incisors were used, and brackets of different brands were bonded to teeth. Seven different bracket brands were tested (Morelli^TM^, American Orthodontics^TM^, TP Orthodontics^TM^, Abzil-3M^TM^, Orthometric^TM^, Tecnident^TM^ and UNIDEN^TM^). Twenty-four hours after bonding, shear bond strength test was performed; and after debonding, the ARI was determined by using an optical microscope at a 10-fold increase.

**Results:**

Mean shear bond strength values ranged from 3.845 ± 3.997 (Morelli^TM^) to 9.871 ± 5.106 MPa (Tecnident^TM^). The majority of the ARI index scores was 0 and 1.

**Conclusion:**

Among the evaluated brackets, the one with the lowest mean shear bond strength values was Morelli^TM^. General evaluation of groups indicated that a greater number of bond failure occurred at the enamel/adhesive interface.

## RESUMO

**Introdução:**

atualmente, há uma grande diversidade de braquetes ortodônticos no mercado brasileiro, e a avaliação desses é fundamental para que os profissionais conheçam suas propriedades e possam qualificar a sua escolha.

**Objetivo:**

avaliar o desempenho de diferentes braquetes metálicos - com diferentes características de base -, por meio da resistência de união e do Índice de Adesivo Remanescente (IAR).

**Material e Métodos:**

braquetes de sete marcas distintas foram testados (Morelli^®^, American Orthodontics^®^, TP Orthodontics^®^, Abzil-3M^®^, Orthometric^®^, Tecnident^®^ e UNIDEN^®^). Os braquetes foram colados em incisivos bovinos totalizando 105 corpos de prova. O teste de resistência ao cisalhamento foi realizado 24h após a colagem e, em seguida, foi avaliado o IAR, por meio do uso de um microscópio óptico, em aumento de 10 vezes.

**Resultados:**

a média dos valores de resistência de união variou entre 3,845 ± 3,997 MPa (Morelli^®^) e 9,871 ± 5,106 MPa (Tecnident^®^). A maioria dos escores do IAR foi de 0 e 1.

**Conclusão:**

entre os braquetes avaliados, o que obteve a menor média de resistência de união foi o Morelli^®^. A avaliação geral dos grupos indicou maior número de falhas de colagem na interface esmalte/adesivo.

## INTRODUCTION

With the enamel-etching technique introduced by Buonore,^1^ direct bonding of orthodontic accessories, which used to be welded to metal bands, became possible.^1^ Direct bracket bonding to dental enamel has been studied over the years, and evaluation of bonding systems and different types of enamel surface preparations prior to bonding^2^ ^-^ ^5^ has been conducted in an attempt to improve and obtain adequate bond strength in Orthodontics.^2^ ^,^ ^3^ ^,^ ^6^ ^-^ ^9^

There is a wide variation in methods and results of shear bond strength tests in the literature,^10^ ^,^ ^11^ ^,^ ^12^ which makes the comparison to some of these studies difficult and enhances the need for new and methodologically standardized studies.^11^ ^,^ ^13^ Besides the bonding material used and the enamel surface preparation,^3^ the type of bracket and its base design influences bond strength^14^ which has to be strong enough to allow the normal course of orthodontic treatment and to resist masticatory efforts.^15^ ^,^ ^16^

According to previously reported literature, adequate shear bond strength for orthodontic bonding should be from 5.6 to 7.8 MPa.^17^ It is important to remember that high bond strength values are potentially dangerous, as they may cause enamel fractures during debonding.^10^ ^,^ ^16^ ^,^ ^18^ In order to improve adhesive retention to orthodontic metal brackets, different chemical and mechanical retentive base configurations have been proposed,^19^ and many different brackets and their base types have been evaluated.^14^ ^,^ ^15^ ^,^ ^19^ ^-^ ^23^

Orthodontic treatment success majorly depends on the correct application of sustained forces applied to teeth via brackets.^24^ Since these brackets play a significant role in the correction of malocclusion, their evaluation is mandatory. This study aims to evaluate the bond strength and the amount of adhesive left on the enamel (Adhesive Remnant Index [ARI]) after bonding metal brackets with seven different brands, so as to provide useful scientific information that may help clinicians to choose which bracket to use. 

## METHODS

The study was performed at *Universidade Federal do Rio Grande do Sul*, School of Dentistry, Dental Material Laboratory and at the Biomaterial Laboratory of the School of Engineering of the same university. A sample of 105 permanent bovine incisors was selected for this study. The incisors were donated by a certified slaughter-house, all from animals slaughtered for meat consumption and whose teeth would otherwise be discarded. 

In order to meet the inclusion criteria, all bovine teeth had to be permanent incisors with intact buccal enamel and without any cracks. After extraction, the teeth were cleaned with complete removal of the periodontal ligament, and the roots were sectioned at their apical portion. Teeth were stored in distilled water at 5^o^C. The buccal enamel surfaces were standardized with #400 and #600 grain abrasive papers in a polisher under constant water irrigation for 50 seconds per tooth, obtaining flat enamel surfaces. 

**Figure 1:**
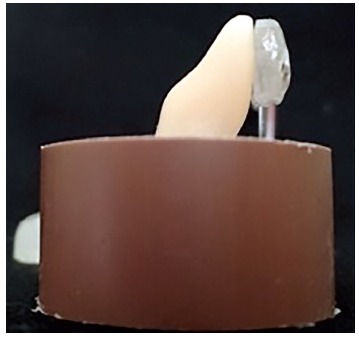
Image of the positioner used to guide the tooth position.

To make the test specimens, a positioner was developed to allow and ensure that the buccal surface of each tooth was perpendicular to the floor (Fig 1). Teeth’s buccal surfaces were positioned as mentioned and the crowns were fixed with wax to the device, with their root portion free to be inserted in a polyvinyl chloride ring with 20-mm diameter and 15-mm height (Amanco^TM^, São Paulo, Brazil). The roots were positioned in the center of the rings, and self-cured acrylic resin (Vipi, Pirassununga, Brazil) was poured onto it.

**Figure 2:**
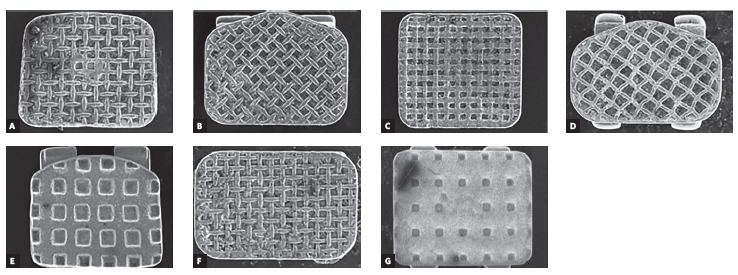
Scanning electron microscopy showing the bracket base type of each group. A) Group 1; B) Group 2; C) Group 3; D) Group 4; E) Group 5; F) Group 6; G) Group 7.

The prepared test specimens were stored in distilled water and maintained at 37 ^o^C for 24 hours to simulate oral temperature. 

Orthodontic stainless steel maxillary central incisor brackets of different brands were bonded to the teeth with Transbond XT (3M Unitek^TM^), following the manufacturer’s instructions. Seven different bracket brands were tested (Morelli^TM^, American Orthodontics^TM^, TP Orthodontics^TM^, Abzil-3M^TM^, Orthometric^TM^, Tecnident^TM^ and UNIDEN^TM^), and all brackets had 0.022-in edgewise standard slots. All teeth were prepared and bonded by the same operator, who was blinded in relation to bracket brand and also calculated all bracket base areas. The brackets used in each group and the type of bracket base retention are presented in Table 1. Bracket bases were analyzed by scanning electron microscopy (SEM) and are presented in Figure 1.

**Table 1:** Brackets used in each group and type of base retention.

Group	Bracket	Bracket base retention
1	UNIDEN™	Relatively small pin-shaped metallic proeminences
2	Morelli™	Mesh base with relatively small spacing
3	Orthometric™	Mesh base with relatively large spacing
4	American Orthodontics™	Mesh base with relatively small spacing
5	TP Orthodontics™	Mesh base with relatively small spacing
6	Tecnident™	Relatively large pin-shaped metallic proeminences
7	Abzil-3M™	Mesh base with relatively small spacing

Before bonding, the teeth were cleaned and polished with rubber prophylactic cups (Viking, KG Sorensen, Barueri, Brazil) and fluoride-free pumice (S.S. White, Juiz de Fora, Brazil), then rinsed with water for 10 seconds, to remove any pumice debris, and dried for the same time. Thereafter 37% phosphoric acid gel was applied to the enamel buccal surface of each tooth for 30 seconds. The teeth were then rinsed with a water spray for 10 seconds and dried with an oil-free, water-free air source for 3 seconds at a 15-cm distance.

Transbond TM XT (3M Unitek^TM^) primer-adhesive was applied on the etched surfaces and Transbond XT (3M Unitek^TM^) composite resin was placed on each bracket base. The brackets were then properly positioned on the buccal surfaces of teeth and subjected to a 454-g force with a Gillmore needle for standardization. Excess resin was removed with the aid of an explorer. 

The composite resin was light-cured for 20 seconds (10 seconds mesial and 10 seconds distal to the bracket) with a halogen light with intensity around 600 mW/cm² at a distance of 5 mm, according to the manufacturer’s instructions.

Twenty-four hours after bonding, shear bond strength test of all specimens was performed in a Universal Testing Machine (Instron Corporation, Canton, USA) with a load cell of 500 N and crosshead speed of 0.5 mm/min. All specimens were tested by the same operator. The results of each test were given in MPa and recorded by a computer that was connected to the testing machine. 

After debonding, the enamel surface of each tooth was examined to have the fracture pattern accessed, and the Adhesive Remnant Index (ARI) was determined using an optical microscope under 10X magnification. All teeth were analyzed by the same observer. The ARI, as proposed by Artün and Bergland,^25^ was used to classify the enamel surface after debonding, according to the following scores: score 0, no composite resin left on the tooth; score 1, less than half of composite resin left on the tooth; score 2, more than half of composite resin left on the tooth; score 3, all composite resin left on the tooth with distinct impression of the bracket base. 

The bracket/adhesive interface can be considered the most favorable failure site for safe debonding, leaving most of the adhesive on the enamel surface,^26^ ^,^ ^27^ as seen in scores 2 and 3. This interface can be considered safe, since there is less chance of enamel fracture.^26^ ^,^ ^27^

Sample size calculation and statistical analysis were performed with the aid of SigmaPlot 11.0 software (California, USA). The parameters adopted were: significance level set at 5%, power test of 80%, mean shear bond strength value of 12.96 ± 3.0 MPa^28^ and effect size equal to 1.^11^ ^,^ ^29^ Data were analyzed for normal distribution by means of Kolmogorov-Smirnov test, then submitted to one-way analysis of variance and Tukey’s multiple comparison test. Data from ARI score were submitted to Kruskal-Wallis test. The significance level used was 95%.

## RESULTS

Mean values of shear bond strength for each Group are listed in Table 2.

**Table 2:** Shear bond strength values shown by each group.

Group	Bracket	n	Mean (MPa)	Standard deviation
1	UNIDEN™	15	6.696^AB^	± 3.450
2	Morelli™	15	3.845^B^	± 3.997
3	Orthometric™	15	9.388^A^	± 5.237
4	American Orthodontics™	15	6.942^AB^	± 5.277
5	TP Orthodontics™	15	5.479^AB^	± 2.809
6	Tecnident™	15	9.871^A^	± 5.106
7	Abzil-3M™	15	6.509^AB^	± 3.528

Different superscript capital letters show statistically significant difference (*p* < 0.05).

Group 6 (Tecnident^TM^) presented the highest mean value for shear bond strength, with statistically significant difference from Group 2 (Morelli^TM^) (*p*= 0.004). The second highest mean bond strength value was obtained by Group 3 (Orthometric^TM^), with statistically significant difference from Group 2 (*p*= 0.011). Although Group 2 obtained the lowest mean shear bond strength value, it was not statistically different from Groups 1, 4, 5 and 7.

The optical microscope analysis under 10X magnification after debonding did not reveal any fractures or cracks of the enamel surfaces. 

Evaluation of the Adhesive Remnant Index (ARI) by Kruskal-Wallis test showed statistically significant difference in the distribution of scores between Groups (*p*< 0.001), specially between Groups 7 and 5 and 7 and 1 (Dunn’s *post-hoc* test). The scores were analyzed at each Group and are shown in Table 3. 

**Table 3:** ARI scores shown by each group.

Groups	ARI			
	0	1	2	3
1^A^	0	4	1	10
2^AB^	4	6	1	4
3^AB^	1	12	2	0
4^AB^	1	8	5	1
5^A^	3	5	1	6
6^AB^	3	8	0	4
7^B^	12	1	0	2

Different superscript capital letters show statistically significant difference (*p* < 0.01).

## DISCUSSION

The results for shear bond strength found in this study ranged from 3.845 ± 3.997 MPa in Group 2 to 9.871 ± 5.106 MPa in Group 6, and were similar to results previously reported.^8^ ^,^ ^15^ ^,^ ^23^

ARI evaluation showed a higher number of scores 0 and 1, except for Group 1, which had a higher number of scores 3. This indicates that the tested sample showed a greater number of bond failures occurring at the enamel/adhesive interface, which is consonant with other reports in the literature.^15^ ^,^ ^16^ These low ARI scores (0 and 1) have been considered favorable by some authors,^6^ ^,^ ^30^ ^,^ ^31^ since there is less adhesive to remove from the tooth surface and, thus, less risk of iatrogenic damage during enamel polishing. Studies have been conducted over this matter, since the literature contains conflicting reports of whether low ARI scores are desirable or not.^27^

A direct correlation between ARI and shear bond strength has been shown.^27^ High ARI scores have been associated with higher bond strengths.^27^ Considering the new evidence about enamel polishing and adhesive removal after debonding, which shows that specific finishing burs can remove the adhesive without damaging the tooth surface,^27^ high ARI scores (2 an 3) - associated with higher bond strengths - may be desired in Orthodontics. It must be considered that the risk of enamel fracture is not exclusively dictated by bond strength; since surface conditioning and debonding techniques can also have great influence.^18^ Fleischmann et al^15^ also found the lowest mean shear bond strength value for Morelli^TM^ Edgewise Standard central incisor bracket, obtaining 3.81 ± 3.56 MPa, which was similar to this study, despite the bonding agent being different (Fill Magic Orthodontic/Magic Bond - Vigodent^TM^).

Bond strength of orthodontic brackets depends on many variables, such as: material and surface structure of the bracket, type of bonding agent used and quality of the enamel.^22^ Additionally, some aspects of the experimental condition can also play a significant roll.

Finnema et al^11^ observed, throughout a meta-analysis, that higher curing time leads to stronger bond strength. The authors found that each additional second of light-curing increased *in vitro* bond strength by 0.077 MPa, but they were not able to find the optimal curing time for bonding. A curing time of 20 seconds adopted in the present study was determined by the manufacturer of Transbond XT bonding system. 

There has been many investigations over the influence of different bracket base designs on bond strength.^19^ ^,^ ^21^ ^-^ ^24^ In order to improve adhesive retention to metal bases, some modifications have been suggested. Mechanical retention can be enhanced by placing undercuts in the bracket bases by welding different diameter wires to the bracket base or by altering the mesh design.^19^

The brackets used in the present study were all stainless steel with mechanical retentive bases, and each type of bracket base retention is described in Table 1. The different mean values for bond strength obtained by the groups of this study indicate that different bracket base designs behave differently under the same test conditions.

It has been suggested that larger bracket bases provide stronger bond strength.^23^ This was not confirmed by the present study, as the highest mean bond strength values (9.39 ± 5.24 MPa and 9.87 ± 5.11 MPa) were obtained by Groups 3 and 6, both of which had the smaller bracket bases; in contrast to Group 2, which had the larger bases and obtained the lowest mean value for bond strength (3.85 ± 3.997 MPa). This suggests that, although the bracket base area may influence bond strength, the type of bracket base design may have an important influence over adhesion to the enamel.

The highest mean shear bond strength values were obtained by Group 6, which had a bracket base with large pin-shaped prominences for retention, similar to Group 1. This kind of retentive base was associated with high bond strength values in a previous study.^14^

The fact that Group 1 had similar retention to Group 6, but showed lower bond strength results - although with no statistical difference -, can be associated with the fact that Group 1 presented a bracket base design with prominences of small size and the presence of welding points. The existence of these welding points has been associated with lower retentiveness, which may reduce the values of bond strength.^19^ ^,^ ^32^

In relation to mesh-type bracket base designs, it has been suggested that larger mesh spacing increases bond strength because the bracket area for resin penetration is larger.^23^ This finding is in agreement with the results from our study, as the strongest bond strength within brackets with mesh-type bases (9.39 ± 5.24 MPa) was found in Group 3, which had the largest mesh spacing base (Fig 1).

Most groups in the present study showed bond failures at the enamel-adhesive interface, which has been considered desirable by some authors,^6^ ^,^ ^30^ ^,^ ^31^ since this results in less adhesive to remove from the enamel surface after debonding. In addition to longer chair time, residual adhesive removal from the tooth surface can also cause surface scratches, cracking and loss of sound enamel.^31^

The determination of a clinical acceptable value for orthodontic bond strength of 6 to 8 MPa, as recommended by Reynolds,^17^ has been widely reported in the literature,^33^ ^,^ ^34^ and bond strengths over 10 MPa have been associated with enlarged risk of enamel fracture during debonding.^35^ However, these precise values have also been criticized^11^ ^,^ ^36^ because there is no scientific evidence that it would be adequate for clinical use.^11^

Eliades and Bourael^36^ stated that these bond strength values are not precise, being based on an estimate of load applied during mechanotherapy, with undefined margin of safety, and not taking into account the aging factor of the material and the stresses developed during mastication.

In order to obtain clinically relevant results from *in vitro* studies, precise simulation of the clinical condition is required. However, this is a difficult and unrealistic goal, considering that many factors are associated *in vivo* ^11^ ^,^ ^12^ ^,^ ^37^ ^,^ ^38^ and the majority of studies over dental adhesives remain *in vitro*.^12^

Similar to what has been recommended for *in vitro* bond strength studies in Orthodontics,^13^ in this study, we used distilled water at 37 °C for 24 hours to store all specimens after bracket bonding. The shear bond strength test was performed with crosshead speed of 0.5 mm/min, the results were expressed in MPa and it was used the ARI as proposed by Artün and Bergland.

Pickett et al^10^ tested an *in vivo* debonding device and compared *in vivo* bond strengths with *in vitro* bond strengths. The authors found that the mean shear bond strength values registered *in vivo* are significantly lower than the ones *in vitro*. 

Although some studies have found higher values for shear bond strength,^11^ ^,^ ^28^ the mean values obtained in the present study did not differ from the results reported in the literature,^8^ ^,^ ^15^ ^,^ ^23^ despite methodological differences existing among them. The findings of this and other *in vitro* studies, however, must be carefully interpreted, since clinical conditions may be significantly different from those of an *in vitro* experiment.^39^ Studies developed *in vivo* or *in situ* may provide additional evidence to these findings, thus enhancing knowledge of bond strength in Orthodontics. 

This study only tested stainless steel brackets bonded with Transbond XT to bovine enamel, and the results cannot be extended to other types of material, such as ceramic brackets, other types of adhesive, different enamel preparations or bonding on different surfaces, such as restorative material. 

## CONCLUSIONS

1) In relation to bond strength, all groups presented similar results, except for Morelli^TM^ brackets, which showed the lowest bond strength results.

2) The majority of the ARI index scores were 0 and 1, with brackets presenting a greater number of bond failures at the enamel/adhesive interface. Although this interface is considered dangerous for the risk of damaging the enamel surface, no damage was observed at teeth after debonding.
